# Exploring the Complexities of Long COVID

**DOI:** 10.3390/v16071060

**Published:** 2024-06-30

**Authors:** Jackson Donald, Shymaa E. Bilasy, Catherine Yang, Ahmed El-Shamy

**Affiliations:** 1 College of Graduate Studies, California Northstate University, 9700 West Taron Drive, Elk Grove, CA 95757, USA; jackson.donald@cnsu.edu (J.D.); catherine.yang@cnsu.edu (C.Y.); 2 College of Dental Medicine, California Northstate University, 9700 West Taron Drive, Elk Grove, CA 95757, USA; shymaa.bilasy@cnsu.edu

**Keywords:** SARS-CoV-2, Long COVID, post COVID-19 condition

## Abstract

Since the emergence of the SARS-CoV-2 virus in 2019, nearly 700 million COVID-19 cases and 7 million deaths have been reported globally. Despite most individuals recovering within four weeks, the Center for Disease Control (CDC) estimates that 7.5% to 41% develop post-acute infection syndrome (PAIS), known as ‘Long COVID’. This review provides current statistics on Long COVID’s prevalence, explores hypotheses concerning epidemiological factors, such as age, gender, comorbidities, initial COVID-19 severity, and vaccine interactions, and delves into potential mechanisms, including immune responses, viral persistence, and gut dysbiosis. Moreover, we conclude that women, advanced age, comorbidities, non-vaccination, and low socioeconomic status all appear to be risk factors. The reasons for these differences are still not fully understood and likely involve a complex relationship between social, genetic, hormonal, and other factors. Furthermore, individuals with Long COVID-19 seem more likely to endure economic hardship due to persistent symptoms. In summary, our findings further illustrate the multifaceted nature of Long COVID and underscore the importance of understanding the epidemiological factors and potential mechanisms needed to develop effective therapeutic strategies and interventions.

## 1. Introduction

The first case of Coronavirus Disease 2019 (COVID-19) surfaced in Wuhan, China, on 08 December 2019. In response to the escalating spread of the SARS-CoV-2 virus, a consequential lockdown was imposed upon the 11 million inhabitants of Wuhan on 23 January 2020 [1]. Despite these efforts, the virus rapidly spread, leading to global proliferation and prompting other nations to institute analogous measures. In the United States, the initial nationwide lockdown was enforced on 15 March 2020 [2].

In the aftermath of the pandemic’s onset, a subset of individuals recovered from the acute phase and began reporting lingering symptoms that encompassed cognitive impairment, fatigue, and a spectrum of other manifestations. In July 2020, Dr. Elisa Perego, an Honorary Research Fellow at the University College London, introduced the term “Long COVID” via a Twitter hashtag, sparking widespread discourse on social media as the cohort of individuals experiencing persistent symptoms following SARS-CoV-2 infection, colloquially referred to as “Long-Haulers”, shared their evolving symptomatology [3]. Several research centers detailed the long-term repercussions of acute SARS-CoV-2 infection [3,4]. As the number of “Long-Haulers” increased, comprehensive efforts were undertaken to delineate and define this emerging medical phenomenon. The Centers for Disease Control and Prevention (CDC) and the World Health Organization (WHO) independently released their respective definitions in response to the evolving landscape of research and clinical observations [5,6]. 

**Figure 1:**
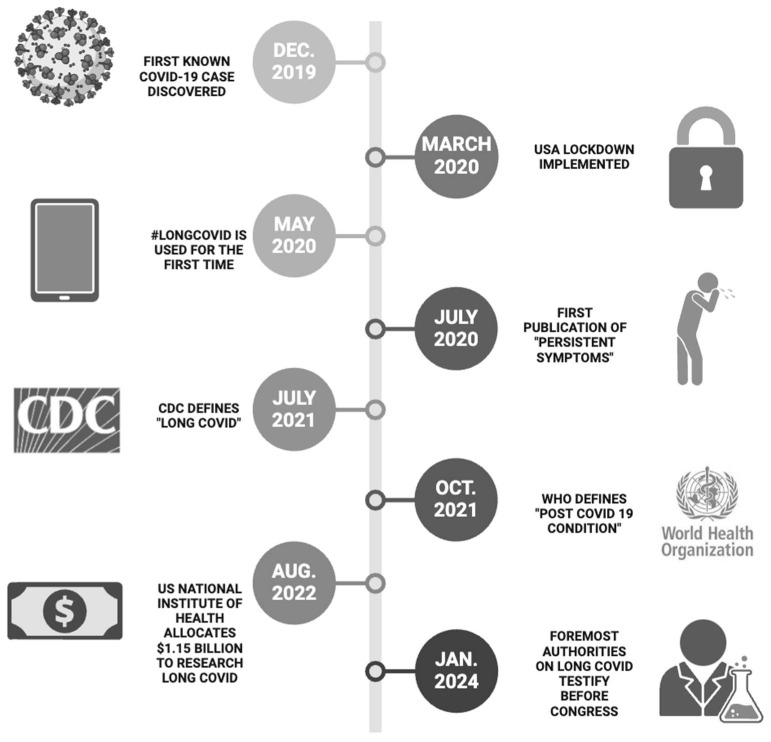
Major milestones in the evolution of Long COVID. A representative timeline for major events, including the emergence of COVID-19, lockdowns, initial reports of Long COVID, and recognition by major organizations, including the WHO and CDC. The attention gained aimed to establish a comprehensive definition, funding, and future allocations, engaging the United States Congress.

The CDC officially termed the condition “Long COVID”, characterizing it as “...a patient-created term broadly defined as signs, symptoms, and conditions that persist or emerge after the initial SARS-CoV-2 infection”. These manifestations are endured for four weeks or more beyond the initial phase of infection [5]. Meanwhile, WHO denominated the condition as the “Post COVID-19 Condition”, delineating it as the continuation or development of new symptoms three months after the initial SARS-CoV-2 infection, and these symptoms can be endured for at least two months without other alternative explanations [6]. Additionally, the National Health Service (NHS) in Great Britain opted for the nomenclature “Post COVID syndrome”, characterizing it as “signs and symptoms that manifest during or after COVID-19 and persist for more than 12 weeks, devoid of an alternative diagnosis [7]. In August 2022, the National Institute of Health (NIH) allocated USD 1.15 billion to Long COVID research (Figure 1). 

To date, the medical community struggles with the lack of a universally agreed-upon nomenclature for a condition known by various names, including Long COVID, post-COVID conditions, post-acute sequelae of SARS-CoV-2 (PASC), chronic COVID-19, ongoing symptomatic COVID-19, post-COVID-19 persistent symptoms, and post COVID-19 syndrome [8]. The significance of assigning a name to a medical condition is underscored by the recognition that language holds consequential implications. Furthermore, researchers contend that the nomenclature assigned carries implicit assumptions about the underlying physiology of the condition. Words such as ‘chronic’, ‘post’, and ‘syndrome’ may inadvertently contribute to delegitimizing the lived experiences of individuals grappling with the repercussions of the ailment. The lack of a definitive definition of Long COVID is attributed to the extensive spectrum of symptoms impacting various organ systems, necessitating the establishment of a comprehensive definition that goes beyond merely cataloging symptoms and instead holistically assesses the patient’s condition. Researchers emphasize the crucial need for a consensus definition, underscoring its pivotal role in ensuring accurate and timely diagnosis for affected patients [9]. This term should be an encompassing term that accurately reflects the condition while avoiding inadvertently stigmatizing the profound impact on those affected [10]. 

Once a consensus definition is established, it lays the foundation for an objective and standardized approach to diagnosis within clinical settings, promoting clarity and precision in patient care. In this study, we aim to summarize the major hallmarks of Long COVID.

## 2. Major Findings

### 2.1. Overview of Long COVID

#### 2.1.1. Symptoms and Prevalence

**Figure 2:**
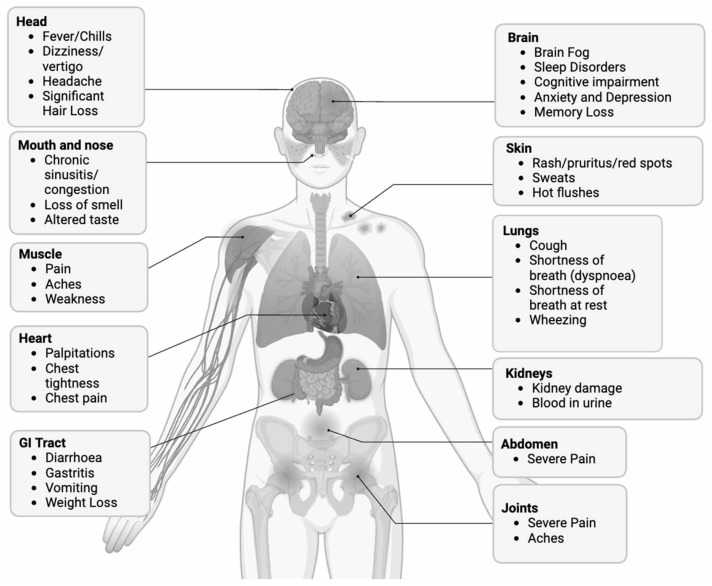
Long COVID symptoms. A representative diagram of Long COVID symptoms. All organ systems appear to be affected, including the heart, mouth/nose, muscle, head, gastrointestinal tract, brain, skin, lungs, kidneys, abdomen, and joints.

**Figure 3:**
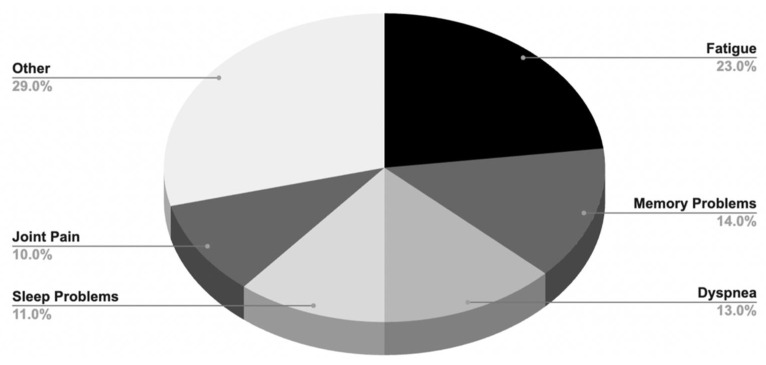
Most prevalent Long COVID symptoms. A pie chart representing the most prevalent five symptoms of Long COVID.

Over 200 symptoms encompassing most organ systems are associated with Long COVID (Figure 2) [9]. Quantifying the prevalence of Long COVID symptoms has posed a significant challenge, owing to the heterogeneous nature of the condition and the complex array of symptoms associated with it. In a meta-analysis encompassing forty-one studies, researchers identified fatigue (23%), memory problems (14%), dyspnea (13%), sleep problems (11%), and joint pain (10%) as prominent features of Long COVID (Figure 3) [11]. A prospective longitudinal study analyzing 9764 patients identified thirty-seven symptoms with a frequency exceeding 2.5% [12]. Similarly, the prevalence of Long COVID exhibited considerable variability. The WHO estimated that approximately 10–20% of individuals infected with SARS-CoV-2 could develop Long COVID [13], whereas a Scottish nationwide cohort study of 198,096 SARS-CoV-2 adult patients demonstrated that up to 64.5% of patients reported at least one symptom six months post-SARS-CoV-2 infection [14]. The Office for National Statistics estimated that 3.1% of the population, or around 2 million individuals, have experienced Long COVID [15]. The Household Pulse Survey, conducted by the United States Census Bureau, estimated that 7.5% of adults (1 in 13) exhibited Long COVID symptoms persisting for three or more months [16]. Notably, the absence of objective measures for symptoms often leaves practitioners relying on diagnosis by exclusion, which necessitates the elimination of other diseases with similar differentials before arriving at a Long COVID diagnosis. This subjective nature of diagnosis introduces challenges like the mislabeling of Neurological Dysfunction in Long COVID as Functional Neurological Disorder (FND), leading to erroneous exclusion from Long COVID care for affected patients [17]. Moreover, the global landscape for Long COVID data remains incomplete. Not every country systematically tests for Long COVID, resulting in a dearth of prevalence data, and government databases tracking Long COVID are sparse [18].

#### 2.1.2. Long COVID Duration

The duration of Long COVID is still unknown and varies widely among national databases and studies. Data from the United Kingdom’s Office of National Statistics suggests that among the 1.9 million UK citizens experiencing Long COVID, nearly 41% were still experiencing at least one symptom after one year [19]. In comparison, among the 968 adults with confirmed Long COVID, 85% had at least one symptom after one year in a French cohort study [20]. Discrepancies can be likely attributed to the vast symptom profiles of Long COVID and the differences in severity among patients.

### 2.2. Demographic Findings

#### 2.2.1. Female Sex

**Figure 4:**
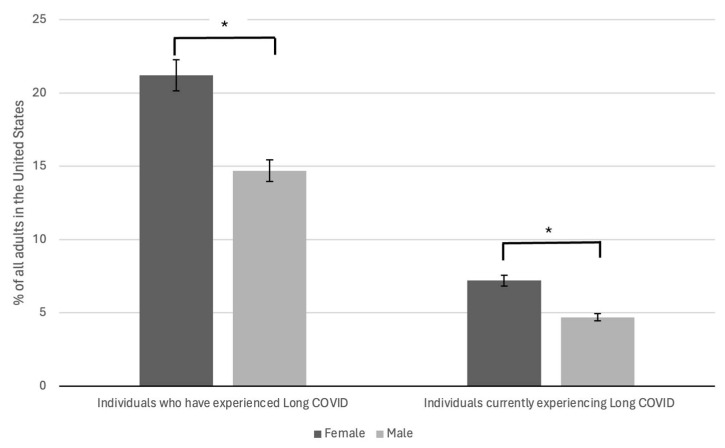
Gender disparities of Long COVID in the US. A bar graph depicting the gender of Long COVID adult patients. Compared to males, the number of Long COVID patients was significantly higher in females (21.2% vs. 14.7%, * *p* < 0.05). Additionally, more females (7.2% vs. 4.7%, * *p* < 0.05)) currently have Long COVID compared to males.

Accumulating evidence indicates that the female sex may be a significant risk factor for developing Long COVID. According to the United States Census Bureau’s Household Pulse Survey, approximately 21.2% of SARS-COV-2-positive females developed Long COVID compared to 14.7% of males (Figure 4) [16].

Notably, perimenopausal females (around 50 years old) had the highest risk, suggesting a possible hormonal influence [21]. Estrogen and progesterone receptors are expressed in most immune cells [22,23]. Prior research demonstrated that estrogen has an immunomodulatory function with both pro-inflammatory and anti-inflammatory functions depending on several factors that include the immune stimuli and the female’s reproductive status [24]. Progesterone can also modulate the immune response with mainly anti-inflammatory functions [25]. In a randomized controlled pilot trial, the addition of progesterone to the standard of care treatment protocol improved the severity of COVID-19 outcomes in male patients [26]. Due to their immunomodulatory effects, the role of sex hormones was investigated as a potential explanation for the observed sex disparities in Long COVID. In the context of COVID-19, angiotensin-converting enzyme 2 (ACE2), the main viral receptor, and type 2 transmembrane serine protease (TMPRSS2), the viral entry facilitator, appeared to be regulated by sex steroids [27]. Kalidhindi et al., demonstrated that ACE2 expression was significantly upregulated by testosterone in primary isolated human airway smooth muscle cells, while estrogen downregulated ACE2 in differentiated airway epithelial cells [28]. Testosterone was also believed to have anti-inflammatory properties, making it a subject of interest in clinical trials [29]. However, the exact impact of steroid hormones is not yet fully understood as they each appear to have both protective and deleterious effects. 

Nevertheless, differences in immune responses between males and females are well-established, with studies suggesting that women exhibit a more effective immune response. This is reflected by the lower mortality rates, reduced inflammation, higher lymphocyte counts, and faster antibody responses seen in women with acute COVID-19 [30]. While an effective initial immune response appears beneficial during acute infection, it may enhance susceptibility to post-infection complications like Long COVID. Non-inflammatory persistent viral reservoirs may trigger chronic inflammation in females and predispose them to virus-induced autoimmunity [31,32]. X-chromosomes have the largest number of immune-associated genes. Although one chromosome of the X chromosomes usually undergoes silencing, incomplete inactivation has been linked to an increased risk of auto-immune diseases [33,34]. 

The impact of Long COVID on pregnancy is still not fully understood. Recent studies have shown a lower incidence of Long COVID among pregnant females compared to non-pregnant females, suggesting a possible immunological advantage [35]. Further investigation into the incidence of Long COVID among pregnant females is needed.

#### 2.2.2. Comorbidities

Several comorbidities, including but not limited to asthma, chronic obstructive pulmonary disorder (COPD), diabetes, immunosuppressive disorders, and ischemic heart disease, have emerged as significant risk factors associated with the development of Long COVID [36]. The association between Long COVID and these comorbidities can be attributed to a common metabolic proinflammatory process that triggers chronic inflammation and leads to a cascade of associated symptoms. Furthermore, this can indicate a mutual biological mechanism for immune system dysregulation [37]. This phenomenon not only sheds light on the complexity of Long COVID but also emphasizes the need for a comprehensive understanding of the underlying pathophysiology. 

#### 2.2.3. Age-Related Risk Factors

Initially, Long COVID was thought to have a lower incidence in children and adolescents compared to adults. However, emerging information indicates a similar prevalence across non-elderly age groups. The complexity of Long COVID is compounded in younger individuals, as its manifestation in this demographic group appears to be diverse, presenting unique challenges for researchers [38]. The complexity of Long COVID manifestations in children and adolescents can be attributed to several factors, including the limited vocabulary of younger individuals, which may hinder their ability to articulate and report symptoms accurately. Furthermore, subtle and misattributed symptoms, as well as the dynamic nature of the symptoms, can complicate the recognition and diagnosis of Long COVID in this population [39]. In addition, due to their typically lower viral loads, children have a higher prevalence of false-negative COVID-19 PCR tests [40]. Therefore, exploring other alternative diagnostic approaches to unveil the true extent of Long COVID in the pediatric population is needed. Multisystem inflammatory syndrome in children (MIS-C) is a rare yet serious condition linked to COVID-19. It typically occurs 2–6 weeks after SARS-CoV-2 infection and causes inflammation in multiple organ systems [41]. Understanding its mechanisms could provide valuable insights into understanding Long COVID. 

The elevated risk of Long COVID with advanced age is often attributed to factors including the increased risk of mortality, age-related immune dysregulation, the underreporting of symptoms in older adults, the misattribution of Long COVID to other health conditions, and higher vaccination rates [42,43]. A comparison between individuals with COVID-19 and non-infected controls from the US Department of Veterans Affairs highlighted how those individuals over 60 years old experienced a higher incidence of multiple symptoms [42,44]. This susceptibility among the elderly may be attributed to COVID-19 exacerbating pre-existing health conditions.

#### 2.2.4. Socioeconomic Status

Long COVID appears to disproportionately impact individuals with lower socioeconomic status, as indicated by a community-based survey encompassing over 200,000 working-age adults in the United Kingdom. This survey revealed that participants residing in the most deprived areas faced, on average, a 46% higher likelihood of experiencing Long COVID compared to their counterparts residing in the least deprived areas [45]. Barriers to healthcare access, including challenges in testing, treatment, and follow-up care, could contribute to the delayed diagnosis and management of acute COVID. Combined, this can elevate the risk of developing Long COVID. In addition, individuals with lower socioeconomic status often have a higher prevalence of other comorbidities like diabetes, hypertension, and other respiratory conditions, which may increase their susceptibility to Long COVID [46]. Occupational factors can also play a role, with jobs commonly associated with lower socioeconomic status carrying a higher risk of virus exposure [47].

Likewise, individuals grappling with Long COVID frequently experience financial hardships. A cohort study involving nearly 7000 families demonstrated increased economic hardship among Long COVID patients compared to those without a history of COVID-19 infection [48]. This could be attributed to a higher risk of job loss or accumulating substantial medical bills in Long COVID case patients. Although Long COVID is officially recognized as a disability under the Americans with Disabilities Act (ADA; Section 504 and Section 1557), evaluating individual claims requires a thorough assessment. Additionally, the Social Security Disability Insurance (SSDI) program is designed for disabilities lasting longer than 12 months, posing a possible challenge for Long COVID patients who may not have experienced symptoms for a sufficient duration [49].

#### 2.2.5. Vaccine Status

Vaccination against SARS-CoV-2 has emerged as a pivotal measure in decreasing the risk of Long COVID [50]. In comparison to unvaccinated individuals, the prevalence of Long COVID was 40–60% lower among vaccinated adults, defined as those who had completed an initial vaccine series at least 14 days before the onset of COVID-19 symptoms [51]. Various factors can contribute to the efficacy of vaccines in preventing Long COVID. COVID-19 vaccines stimulate the immune system to mount a robust and targeted response against the virus, facilitating efficient viral clearance and lowering the likelihood of persistent viral reservoirs and long-term complications. COVID-19 vaccines were reported to be effective at averting severe illness, hospitalization, and mortality. By mitigating the severity of primary infection, vaccines play a crucial role in reducing the likelihood of enduring complications associated with severe cases of COVID-19. Additionally, vaccinated individuals who contract COVID-19 usually have lower viral loads, which mitigate the severity of the illness, decrease transmissibility, and can reduce the incidence of Long COVID [52].

The cumulative impact of successive vaccine doses is noteworthy. A Swedish population-based study demonstrated that multiple doses of SARS-CoV-2 vaccines, including BNT162b2 (Pfizer-BioNTech), mRNA-1273 (Moderna), AZD1222 (Oxford-AstraZeneca), Ad26.COV2.S (Janssen/Johnson & Johnson), and NVX-CoV2373 (Novavax), significantly reduced the risk of Long COVID [53]. One vaccine dose reduced Long COVID risk by 21%, two doses by 59%, and three doses by a substantial 73%, emphasizing the importance of additional doses in bolstering protection. Studies evaluating the efficacy of different vaccine types were conducted. Each vaccine type demonstrated a capacity to decrease the risk of Long COVID with varying levels of effectiveness. A staggered cohort study using primary care records from the UK, Spain, and Estonia compared different vaccines and found a slightly higher vaccine effectiveness for BNT162b2 (Pfizer-BioNTech) compared to AZD1222 (Oxford-AstraZeneca) in preventing persistent COVID-19 symptoms [54]. These findings underscore the broader effectiveness of diverse vaccines in mitigating the impact of Long COVID.

### 2.3. Immunological Signature and Biomarkers

To unravel intricate details of host–pathogen interactions, disease progression, and potential therapeutic interventions, a comprehensive immunological profile of Long COVID was established [32]. Analysis of the peripheral blood mononuclear cell (PBMC) populations between individuals with Long COVID and demographically matched control groups revealed striking immunological variations. Long COVID patients exhibited elevated levels of non-conventional monocytes, double-negative B-cells, IL-4/IL-6-secreting CD4+ T cells, and antibodies specific to SARS-CoV-2, the Epstein–Barr virus, and varicella-zoster virus antigens. Conversely, these patients demonstrated diminished levels of conventional dendritic cells, central memory CD4+ T cells, and systemic cortisol [32]. In addition, cytokines and particularly increased levels of interferon-γ (IFN-γ) from the PBMCs were observed in Long COVID patients [55].

Interestingly, a potential association between increased pro-inflammatory cytokines like TNF-α and IL-6 and decreased levels of the anti-inflammatory IL-10 in Long COVID was observed [56,57]. This indicates a possible link between these cytokines and persistent Long COVID symptoms. In contrast, reduced circulating serotonin levels were demonstrated in Long COVID patients, implying a potential link to diminished tryptophan uptake due to increased type I interferons [58]. Likewise, reduced systemic cortisol levels without the compensatory release of adrenocorticotropic hormone from the pituitary gland were observed in Long COVID patients [32]. These results indicated the potential impairment in the hypothalamic–pituitary axis. Interestingly, cortisol levels were identified as a strong predictor of Long COVID status [32].

### 2.4. Pathophysiologic Considerations

The pathophysiological mechanism for Long COVID has been the focus of several studies. The persistence of the SARS-CoV-2 virus or its remnants involves intricate modulation of the viral and cellular gene expression and modulation of the host immune response, which may lead to chronic inflammation and sustained symptoms [59]. To enable their persistence, viruses employ different immune evasion strategies, including antigenic variation, the downregulation of immune components, and establishment in immune-privileged sites [59]. The surveillance of national infection data estimated that a considerable number of patients maintain intermittent high viral loads for at least two months (1 in 200 infections to 1 in 1000 infections) [60]. Moreover, SARS-CoV-2 could be detected in the feces and cerebrospinal fluid months after infection [61].

Accumulating evidence suggests the emergence of immune dysregulation and autoimmune diseases following SARS-CoV-2 infection [31]. Both host and genetic factors contribute to the development of autoimmunity. Although the autoantibody reactivities did not differ significantly between Long COVID and the control groups, research studies showed that the elevation of autoantibodies could increase the risk of new-onset autoimmune diseases in Long COVID patients [32,62]. T cells, instrumental in maintaining viral latency, could potentially be compromised by SARS-CoV-2, which may lead to the activation of latent viruses [63]. Interestingly, prior research showed that 66.7% of Long COVID patients exhibited reactivated Epstein–Barr virus infection [64].

#### 2.4.1. Nervous System

Long COVID patients present with a broad array of neural and cognitive symptoms, including cerebrovascular disorders, peripheral nerve disorders, movement disorders, cognitive impairments, mental health issues, sensory disturbances, and various neurologically related conditions, such as dizziness, somnolence, Guillain–Barré syndrome, encephalitis or encephalopathy, and transverse myelitis [65].

**Figure 5:**
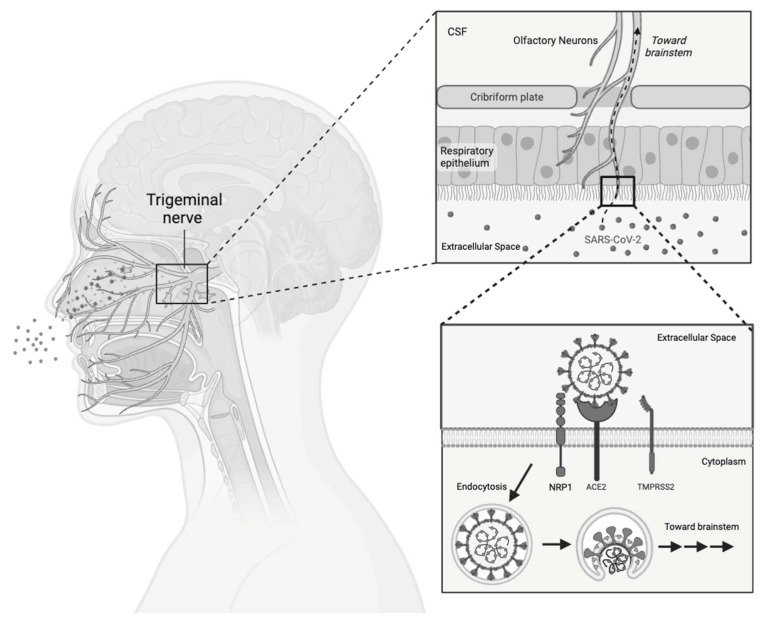
Direct viral invasion through the olfactory bulb. A schematic diagram demonstrating the proposed viral entry through the olfactory bulb. Alongside the ACE-2 receptor, NRP1 stands as an alternative entry point for the virus. SARS-CoV-2 may engage either NRP1 or ACE-2, infiltrating olfactory neurons and utilizing them as a pathway for direct access to the brain.

SARS-CoV-2 is among the neurotropic viruses, with around 80% of hospitalized patients displaying neurological manifestations [66]. In Long COVID, neurological and cognitive manifestations were attributed to the direct viral invasion of the central nervous system (CNS) and/or generalized neuroinflammation. Advocates of direct viral invasion propose potential routes like migration through the nasal cavity or trafficking across the blood–brain barrier [65]. In this context, the olfactory bulb, housing neurons extending into the olfactory mucosa, and the olfactory pathway are theorized as a potential conduit for viral entry into the CNS (Figure 5). ACE-2 and TMPRSS2 are well-established viral receptors that aid in viral entry and infection. The olfactory epithelium has elevated ACE-2 and TMPRSS2 expression and is a well-established viral entry point that can be followed by subsequent migration to the CNS. In particular, the nervus terminalis, characterized by fibers extending from the olfactory epithelium to limbic structures like the hypothalamus, emerges as a potential anatomical route for viral transmission [67]. In neuronal cells, emerging evidence suggests that NRP1 and NRP2 receptors within the olfactory bulb may also act as viral entry points through an intranasal pathway [68].

**Figure 6:**
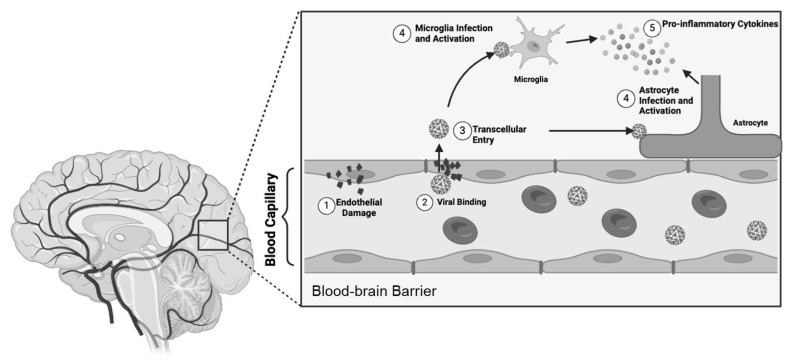
Direct viral invasion across the blood–brain barrier. A schematic diagram demonstrating the proposed pathogenesis across the blood–brain barrier. 1. Viral proteases induce endothelial damage. 2. SARS-CoV-2 binds to ACE-2 receptors. 3. The virus traverses the blood–brain barrier via the transcellular pathway. 4. Astrocytes and microglia undergo direct infection, triggering activation. 5. Activated astrocytes and microglia secrete cytokines, initiating a cascade that further activates the immune cells.

In addition, SARS-CoV-2 protease (Mpro) has been implicated in inducing endothelial cell death and blood–brain barrier disruption which facilitates traversing the microvascular endothelial cells and potentially causes barrier leakage. This breach may facilitate the entry of pro-inflammatory cytokines, fostering a neuroinflammatory state and contributing to the diverse neurological and cognitive manifestations observed in Long COVID (Figure 6) [69].

It is worth mentioning that cerebrospinal fluid analyses from living patients with neuropsychiatric manifestations failed to detect viral RNA [70]. Instead, evidence suggests that immune activation, secondary to autoimmune responses or persistent viral infection in tissue reservoirs, is the primary driver for neurologic manifestation in acute COVID-19. Specifically, cerebrospinal fluid analysis revealed increased interferon-producing dendritic cells, activated monocytes, T and NK cells, as well as increased IL-1 and IL-12 levels. Also, antibodies recognizing SARS-CoV-2 spike protein epitopes cross-reacting with neural antigens and T-cell exhaustion were detected [55]. This leads to a neuroinflammatory state that results in the activation of microglia and astrocytes. Mouse models of mild SARS-CoV-2 infection demonstrated that microglial reactivity was associated with cognitive dysfunction and impaired neurogenesis. Glial cells can release glutamate and reactive oxygen species (ROS), which contribute to the development of neurological manifestations [71]. Furthermore, ATP can act as a pathogen-associated molecular pattern that can activate the release of proinflammatory cytokines and activate the NLRP3 inflammasome [72].

#### 2.4.2. Cardiovascular System

Microvascular impairments have been reported in acute and Long COVID. Patients reported a wide range of cardiovascular symptoms, including palpitations, chest pain, shortness of breath, myocardial injury, heart failure, arrhythmias, vascular injury/thrombosis, and dysautonomia [73]. This underscores a complex interplay between SARS-CoV-2 and the cardiovascular system that may result in potential long-term cardiovascular adverse outcomes. A significant subset of SARS-CoV-2 patients continue to manifest cardiac abnormalities even after recovery [74].

The American College of Cardiology classified COVID-19 patients into two distinct patient subpopulations: those with cardiovascular risk factors or preexisting disease conditions and those with cardiovascular symptoms but lacking evidence of prior cardiovascular conditions. The former group tended to exhibit more severe outcomes, including myocardial dysfunction, ischemia, and inflammation, while the latter group (without preexisting cardiovascular disease) was more prone to chest pain and palpitations [75]. In Long COVID, cardiac symptoms can be caused by the direct invasion of SARS-CoV-2 to the heart muscle [76]. This direct invasion may lead to inflammation and damage to the cardiomyocytes. Furthermore, the immune response triggered by the virus may lead to the initiation of inflammatory processes in the cardiovascular system. For instance, SARS-CoV-2 can contribute to persistent capillary rarefaction even at 18 months post-infection [77]. The reduction in vascular density can be associated with cardiovascular and renal adverse outcomes [78]. Alternatively, SARS-CoV-2 has the potential to induce a hypercoagulable state, leading to the formation of blood clots within coronary arteries and possible ischemic sequelae [79].

Beyond direct viral involvement, immune dysregulation and autonomic dysfunction are proposed mechanisms for Long COVID-related cardiac symptoms [80]. A sustained immune response following the initial infection may sustain inflammation in the cardiovascular system. Additionally, the dysregulation of the autonomic nervous system may worsen cardiovascular adverse outcomes [81]. Understanding the exact mechanism is paramount for developing targeted interventions to address the cardiovascular complications in Long COVID patients.

Postural Orthostatic Tachycardia Syndrome (POTS), a form of orthostatic intolerance, is associated with hypovolemia and compensated by an increase in cardiac output. Approximately 50% of POTS cases were preceded by an acute viral illness, with SARS-CoV-2 identified as one of the potential triggers [82]. It is noteworthy that around 80% of Long COVID patients met the diagnostic criteria for POTS [83]. Therefore, SARS-CoV-2 infection may serve as a potent immune trigger, evoking an autoimmune response and cardiovascular adverse events in susceptible individuals. However, further research is imperative to verify this connection.

#### 2.4.3. Respiratory System

Long COVID has been associated with persistent coughing and shortness of breath. Pneumonia induced by COVID-19 can exacerbate breathing difficulties and inflict alveolar damage. Imaging studies have consistently unveiled pulmonary abnormalities in Long COVID patients [84]. Dissecting the pathophysiological mechanism can be instrumental in formulating targeted interventions to mitigate pulmonary adverse outcomes. This can be partly explained by the persistence of the virus within the lungs. In addition, elevated levels of pro-inflammatory cytokines could potentially contribute to the development of pulmonary fibrosis in Long COVID patients. Together, this emphasizes the intricate relationship between viral persistence and the inflammatory response in the respiratory system [85].

#### 2.4.4. Gut Microbiome Alterations

COVID-19 patients were at a higher risk of developing digestive diseases [86]. Long COVID’s gastrointestinal symptoms encompass abdominal pain, diarrhea, nausea, loss of appetite, and inflammatory bowel disease. Gastrointestinal outcomes may be affected by immune dysfunction, persistent inflammation, dysbiosis, metabolite production, and loss of mucosal integrity. SARS-CoV-2 infection may disrupt the gut microbiome, fostering questions about the role of beneficial or dysbiotic bacteria in prolonged inflammation [87]. Furthermore, damage to the gut epithelium during infection may exacerbate systemic inflammation and increase susceptibility to secondary infections. Interestingly, dysbiosis can adversely affect the immune response in the lungs through the gut–lung axis [88]. Research findings suggest that alterations in the gut microbiome may lead to neurological manifestations like brain fog and cognitive dysfunction in Long COVID patients through the gut–brain axis [89].

### 2.5. Treatment

General treatment guidelines include rehabilitation services for physical limitations, mental health support for psychological impacts, exercise and physical activity, nutritional support, and pharmacological interventions [90]. In 2023, the Food and Drug Administration approved the antiviral agent nirmatrelvir/ritonavir (Paxlovid) for treating mild-to-moderate COVID-19 in adults. The RECOVER Initiative, a USD 1.15 billion research platform aimed at identifying viable treatment options for Long COVID patients, launched its first prospective, randomized study to evaluate nirmatrelvir/ritonavir as a potential treatment for Long COVID [91]. The RECOVER-VITAL study is expected to enroll around 900 participants, with a completion date estimated in fall 2025 [92]. To date, several other studies have investigated the association between Long COVID and nirmatrelvir treatment, but the results were not consistent. An online observational cohort study found no association between nirmatrelvir/ritonavir use and a lower prevalence of patient-reported Long COVID symptoms [93]. Similarly, for Congdon et al., nirmatrelvir treatment was not associated with decreased Long COVID risk [94]. In contrast, Xie et al. reported that nirmatrelvir treatment within five days of positive SARS-CoV-2 diagnosis was associated with decreased risk of developing Long COVID. 

Additionally, a phase 3, randomized, quadruple-blinded placebo-controlled clinical trial assessed Long COVID outcomes in patients receiving metformin, ivermectin, or fluvoxamine. Metformin, an anti-hyperglycemic agent, demonstrated a 41% reduction in Long COVID incidence compared to the placebo, while no significant differences were observed with ivermectin or fluvoxamine [95]. Researchers suggest that metformin may decrease the SARS-CoV-2 viral load in the body by inhibiting the mechanistic target of rapamycin (mTOR), thereby controlling protein translation. 

Interestingly, the opioid antagonist naltrexone and nicotinamide adenine dinucleotide (NAD) have shown benefits for long-term fatigue symptoms of COVID-19 [96]. Naltrexone acts as a glial cell modulator and has been widely used off-label to treat inflammation and autoimmune diseases, such as Crohn’s disease [97]. NAD is a coenzyme essential for metabolism and has been found to decline during infection [98]. Furthermore, the symbiotic drug SIM01, an oral encapsulated formulation of three lyophilized bifidobacteria and prebiotics, has demonstrated the ability to restore gut health. Studies have shown reduced pro-inflammatory markers and relief from multiple Long COVID symptoms, with fecal metagenomic analyses indicating a more diverse microbiota [99]. Nevertheless, there is no FDA-approved single pharmacological therapy guideline for Long COVID. 

## 3. Conclusions

The first case of COVID-19 emerged in Wuhan, China, in December 2019, leading to a global spread and subsequent lockdowns. As the pandemic progressed, a subset of individuals, known as “Long-Haulers”, experienced persistent symptoms post-infection, leading to the term “Long COVID.” Various health organizations have since defined this condition, highlighting the need for a consensus on the definition to ensure accurate diagnosis and patient care. Long COVID is associated with over 200 symptoms with fatigue (23%), memory problems (14%), dyspnea (13%), sleep problems (11%), and joint pain (10%) as common symptoms. 

Accumulating evidence indicates that the female sex, particularly perimenopausal females, may be a significant risk factor for developing Long COVID, with higher prevalence rates compared to males, possibly due to differences in immune responses and hormonal influences. Various comorbidities, such as asthma, COPD, diabetes, and heart disease, elevate the risk of Long COVID through chronic inflammation and immune dysregulation. Although initially thought to be less prevalent in children, Long COVID affects younger populations with diverse symptoms and diagnostic challenges. Older adults show higher susceptibility, often due to pre-existing comorbid conditions. Lower socioeconomic status is linked to a greater likelihood of Long COVID, exacerbated by barriers to healthcare and increased virus exposure. Vaccination significantly reduces the risk of Long COVID, with multiple doses providing cumulative protection by enhancing immune response, reducing viral loads, and preventing severe illness. This underscores the importance of vaccination in mitigating Long COVID’s impact across different demographics.

A comprehensive immunological profile of Long COVID revealed significant variations, including elevated levels of non-conventional monocytes, double-negative B-cells, certain cytokine-secreting CD4+ T cells, and specific antibodies, along with reduced levels of conventional dendritic cells, central memory CD4+ T cells, and systemic cortisol. These findings suggest potential links between persistent Long COVID symptoms and cytokine imbalances, decreased serotonin, and hypothalamic–pituitary axis impairment. The pathophysiology of Long COVID involves the persistence of SARS-CoV-2 or its remnants, leading to chronic inflammation and immune evasion strategies. Immune dysregulation with elevated autoantibodies, which potentially increase the risk of autoimmune diseases, was reported. Investigating the immunological profile allows researchers to gain insight into understanding disease mechanisms, which can be instrumental in diagnosing and treating Long COVID. Long COVID patients exhibit various neurological symptoms attributed to the direct viral invasion of the CNS and generalized neuroinflammation, with potential entry through the olfactory bulb or blood–brain barrier disruption. Cardiovascular symptoms are common and may result from direct viral invasion of the heart muscle, immune dysregulation, or autonomic dysfunction. Persistent respiratory issues and gastrointestinal symptoms are also reported, linked to viral persistence, inflammation, and gut microbiome alterations. 

Long COVID is likely to persist with subsequent COVID-19 infections, significantly impacting patients and the healthcare system. We recommend collaboration between researchers, patients, and clinicians to deepen our understanding of this condition and to develop effective treatments.

## Data Availability

Not applicable.
